# The Impact of Listening to Background Music on Inhibition Control and Prefrontal Cortical Activation in Healthy Older Adults: A Study Using Functional Near-Infrared Spectroscopy

**DOI:** 10.7759/cureus.69445

**Published:** 2024-09-15

**Authors:** Yuka Okumura, Jun Matsumoto-Miyazaki, Yuka Ikegame, Yoshitaka Asano, Masaru Makibayashi, Jun Shinoda, Hirohito Yano

**Affiliations:** 1 Chubu Medical Center for Prolonged Traumatic Brain Dysfunction, Chubu Neurorehabilitation Hospital, Minokamo, JPN; 2 Cardiology and Respirology, Gifu University Graduate School of Medicine, Minokamo, JPN; 3 Clinical Brain Sciences, Gifu University Graduate School of Medicine, Minokamo, JPN; 4 Emergency Medicine, Central Japan International Medical Center, Minokamo, JPN; 5 Neurosurgery, Chubu Neurorehabilitation Hospital, Minokamo, JPN

**Keywords:** aging, background music, functional near-infrared spectroscopy, go/no-go, inhibition control, older adult, prefrontal cortical activation

## Abstract

Introduction

Aging declines executive functions, including attentional function and inhibitory control, which is the ability to inhibit inappropriate or irrelevant responses. Certain types of background music are negatively correlated with cognitive function. The prefrontal network is correlated with task performance related to executive function. This study aimed to assess the impact of listening to background music on inhibition control and prefrontal cortical (PFC) activation measured using functional near-infrared spectroscopy (fNIRS) in healthy older people.

Methods

In total, 59 healthy volunteers, including 32 healthy older and 27 younger individuals (mean age ± standard deviation: 69 ± 7 and 32 ± 8 years, respectively), participated in this study. The participants completed the inhibition control task (the go/no-go task) and a similar task while listening to certain melodies of children’s songs that are popular in Japan. Changes in cerebral blood flow in the PFC during each task were evaluated using multichannel fNIRS. The relative changes in oxygenated hemoglobin (oxy-Hb) levels during the no-go and go tasks under the music and no-music conditions were compared using a paired t-test. Among the channels with a significant difference in oxy-Hb levels during the go/no-go task between the music and no-music conditions in the older group, the correlation between changes in accuracy response and oxy-Hb levels was validated using Pearson’s correlation test.

Results

The task accuracy was significantly reduced under the music condition compared with that under the no-music condition in the older group but not in the younger group. The accuracy reduction was significantly greater in the older group than in the younger group. In older people, the oxy-Hb levels in 20 channels located in the bilateral Broadman area (BA) 9 and BA46 in the dorsolateral prefrontal cortex and the bilateral BA10 in the frontal pole cortex significantly increased during the no-go tasks under the music condition. During the go/no-go task under the music condition, the decline in task accuracy was significantly correlated with increased oxy-Hb levels in six channels located in the bilateral BA10 in older people.

Conclusion

Background music induced the decline of inhibition control and increase of PFC activity in healthy older adults.

## Introduction

Aging leads to a decline in executive functions, including attentional function, inhibitory control, working memory, and dual-task performance [1,2]. In particular, inhibition control, which is the ability to inhibit inappropriate or irrelevant responses, is the core component of executive functions [1]. Because several daily activities are related to response inhibition, reduction in inhibition control may lead to accidents in daily life and impairments in the quality of life of individuals. The decline in the performance of executive function and attention tests, including the go/no-go test and Stroop test, has been reported to be associated with a risk of future falls after two and five years [3,4]. Therefore, it is important to assess the ability of inhibition control of older people. The go/no-go test, in which participants are required to make a response to specific target stimuli (go) but not to the other stimuli (no-go), is widely used to assess attention and response inhibition [5-7].

Currently, music is prevalent in the daily lives of numerous people. Some individuals occasionally listen to music while performing tasks such as driving, studying, cooking, and exercising. There have been both positive and negative reports regarding the effects of music on cognitive function. Some reports have shown that background music has positive effects on cognitive functions. Preferred background music enhances task-focused attentional state on an easy task [8]. Listening to music, which is relaxing, alleviates mental fatigue and reduces attentional control impairment in undergraduate students [5]. A previous report showed that acoustic background music has no detrimental effects on inhibitory function and neural activation, as assessed using the go/no-go task and event-related potentials [7].

However, some studies have shown that listening to background music is negatively associated with cognitive function, including attentional control, concentration, and working memory [9-13]. A previous meta-analysis revealed that background music, compared to no music, negatively affects cognitive functions including reading process and memory [14]. Furthermore, compared with silence, background music can impair visual associative memory performance in healthy older adults but not in younger ones [15]. Music in the operation theater distracts novice surgeons from performing new tasks [10]. Compared with provocative music or silence, some types of music (e.g., relaxing music) can interfere with attentional control [11]. Happy and high-valence background music is also associated with faster response times in selective attention and greater activations of frontal-parietal areas. Meanwhile, sad and low-valence background music is associated with slower responses and greater occipital recruitment [16].

Cerebral hemodynamic changes related to executive function have been investigated using neuroimaging studies. The prefrontal network, including the dorsolateral prefrontal cortex (DLPFC), anterior cingulate cortex, and frontal pole (FP), is associated with task performance related to executive function and attention control, as determined using functional magnetic resonance imaging and positron emission tomography [17,18]. Recently, functional near-infrared spectroscopy (fNIRS), a noninvasive method for measuring prefrontal cortical (PFC) hemodynamic changes, has been used to investigate brain activation during various tasks, including attention control and inhibition control [19-21].

A previous study review revealed that older adults had greater activation or additional recruitment of PFC regions in cognitive task performance, including executive function, than younger ones [22]. Recent studies on the effects of aging on executive function related to driving have shown that older adults commit more errors and exhibit greater PFC activation than younger ones [23,24]. Therefore, they have additional activated brain circuits to compensate for executive functions reduced by aging [23,24]. However, some studies have shown no difference between younger and healthy older adults in terms of the effects of music on attention control performance and brain activation [11,16].

To the best of our knowledge, no studies have investigated the impact of background music on PFC hemodynamics using fNIRS during attention and inhibition control in healthy older adults. This study aimed to examine the impact of listening to background music on inhibition control using the go/no-go task and on PFC activation using fNIRS in older adults. We hypothesized that background music can reduce inhibition control and induce PFC activation in healthy older adults.

## Materials and methods

Design, setting, and ethics approval

This experimental study was conducted at the Chubu Medical Center for Prolonged Traumatic Brain Dysfunction of Chubu Neurorehabilitation Hospital in Minokamo, Japan. It was approved by the Ethics Committee of Kizawa Memorial Hospital, which was renamed to Chubu Neurorehabilitation Hospital on January 1, 2022 (approval number: 2020-003), and was conducted in accordance with the principles of the Declaration of Helsinki.

Participants

Right-handed healthy volunteers (aged 20-40 years (the young group) and 60-80 years (the older group)) were recruited using a poster about the study in Chubu Medical Center for Prolonged Traumatic Brain Dysfunction, Minokamo, Japan. All participants provided written informed consent. The older group underwent the Frontal Assessment Battery, Logical Memory (LM) Ⅰ and II tests of the Wechsler Memory Scale−Revised (WMS-R), and clinical dementia scale (CDR) to confirm the healthy condition without dementia and mild cognitive impairment (MCI). The exclusion criteria were as follows: (1) people with dementia or suspected dementia based on the revised Hasegawa’s Dementia Scale (HDS−R) [25] and Mini-Mental State Examination (MMSE) [26] (<24 points) in older group; (2) people with mild cognitive impairment (MCI) and suspected MCI based on WMS-R LM II (≤8 for 16 years of education, ≤4 for 10−15 years, ≤2 for 0−9 years) and the clinical dementia scale (≥0.5) [27] in older group; (3) those with impaired hearing or color blindness in both groups; (4) those who could not push the button for the go/no-go task; and (5) those with a history of traumatic brain injury, stroke, and other central nervous system lesions in both groups.

Study protocol

The participants in both groups underwent the go/no-go task, which is the inhibition control task comprising instructions using color presentation under the no-music conditions (i.e., silence) and music conditions on the same day. The condition (with or without music) that should be used first was randomly selected using a random number table created by a computer. The interval between the two conditions was 10 minutes, during which the participants rested in the sitting position.

Behavioral data collection (go/no-go task)

**Figure 1:**
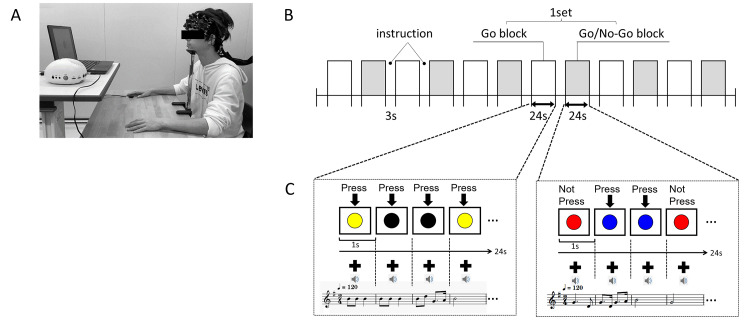
(A) Image of the participants performing the go/no-go task and fNIRS measurements. (B) Experimental protocol. (C) Examples of colored circles that should be pressed or not and music fNIRS: functional near-infrared spectroscopy

The inhibition control function was assessed using the go/no-go task. Based on previous fNIRS studies using the go/no-go task [6], the time course of the go/no-go task was decided. Figure 1 shows the procedure. The go/no-go task was performed using a laptop computer with high-resolution audio (LIFEBOOK, FUJITSU, Kanagawa, Japan) with the stimulus presentation software (PPT2BLUE, Shimazu Co., Kyoto, Japan) linked to the near-infrared spectroscopy (NIRS) system. The laptop monitor was placed approximately 70 cm in front of the participant’s head (Figure 1A).

The procedure comprised six block sets, including alternating go (baseline) block and go/no-go (target) blocks (Figure 1B). After three seconds of display for instructions, each block lasted for 24 seconds, with an overall block-set time of 54 seconds and a total session time of 5.5 minutes. In the go brock (go task), the participants were presented with a random sequence of two types of color circles, and they were instructed to press the button for both circles. In the go/no-go block (no-go task), the participants presented with no-go circles 50% of the time. Thus, they were required to respond to half of the trials (go trials) and not to respond to the other half (no-go trials). The color circles were presented at a frequency of 1 Hz during the go and go/no-go blocks. At the start of each block in the go task, the participants were instructed to press the display for the two colored circles (e.g., yellow and black). In contrast, in the no-go task, the participants were required to press a colored (e.g., blue) circle and not to press the other colored (e.g., red) circle (Figure 1C) on the display. There were three seconds between each block. Each participant underwent a practice block before any measurements to ensure their understanding of the instructions. Each participant was instructed to respond with the index finger of their dominant hand immediately and as correctly as possible according to the instructions when they saw the colored circle. The accurate response rate (%) was calculated as (correct response/total response numbers under the no-go tasks) × 100 in each participant.

Music condition

Under the music condition, the participants were instructed to listen to the background music carefully while undergoing the go/no-go task. The children’s songs used under the music condition were “I have been working on the Railroad,” “Jingle Bells,” “Picnic,” “Mickey Mouse Club March,” “Ah, Lovely Meadows,” and “Tenohira wo Taiyo ni (Put Your Hands up to the Sun),” which were selected from the children’s songbook [28] and were used as supplementary teaching materials in music classes in Japanese elementary schools. These songs were popular in Japan. However, the accurate familiarity among the participants was not quantified.

Melodies without lyrics of those songs were created with piano sounds for 24 measures using music editing software (Domio, TAKABO SOFT). All melodies were set to the 2/4-time signature, and a tempo of 120 beats per minute was used as one measure of one second. Each melody in the mp3 file was divided into one measure and attached to each slide to synchronize the playing of sounds, thereby resulting in the playback of 24 measures of melodies (Figure 1C). The order in which the six melodies were played was randomized for each go block and go/no-go block. The sound of the PC, which presented the color circle for the go/no-go task and the music, was set at a comfortable volume (approximately 50 dB), and the device was placed straightforward from the participants.

fNIRS

Changes in the cerebral blood flow in the prefrontal cortex during each task were observed using a 42-channel NIRS device with three wavelengths of near-infrared light (780, 805, and 380 nm) (SPEERDNIRS, Shimazu Co., Kyoto, Japan). The sampling rate was set at 8.3 Hz. The participants sat in a chair with their chin fixed on a desk, and the NIRS probes were set to cover the forehead and identify the prefrontal cortex. A 3 × 9 multichannel probe holder was used. The holder was attached to the lower anterior lines of the probe holder on the T3-Fpz-T4 line of the international 10-20 system. The source-detector distance was 3 cm. A high-pass filter uses cut-off frequencies of 0.01 Hz to remove baseline drift, and a 0.8-Hz low-pass filter removes heart-beat pulsations. The optical data were analyzed using the modified Beer-Lambert Law [6,29].

This equipment could measure the concentration of oxygenated hemoglobin (oxy-Hb), deoxygenated hemoglobin, and total hemoglobin. Changes in oxy-Hb level can better reflect cortical activity as it directly responds more to cognitive task-related brain activation and is more strongly correlated with blood oxygenation level-dependent signals measured on fMRI [30]. Hence, in this study, oxy-Hb level was used as the primary outcome measure.

We determined the spatial values of the sourced and detector optode locations in each channel of the elastic cap using a three-dimensional digitizing pen [31]. Then, the Montreal Neurological Institute coordinate of each channel was detected using NIRS-SPM ver. 4, and the locations of the gyrus and Broadman area (BA) of each channel were identified using the WFU Pick Atlas ver. 3. The channels located in the DLPFC and FP cortex, which are associated with attention control, inhibition control, and dual-task performance [17-20], were used in the analysis.

The signal time course in each channel was calculated. The raw data in each channel among the individual participants were visually inspected. Moreover, we excluded the trial set with artifact signals, including discontinuous waveforms with a sudden large amplitude in both oxy-Hb and deoxygenated hemoglobin in the same direction by visual observation based on previous fNIRS studies [32,33]. Differences in the integrated values (average in seconds) of Hb signals of the target (average during 4-24 seconds after the go/no-go trial onset) and baseline (average during 0-10 seconds before the go/no-go trial onset (i.e., 14-24 seconds after the go trial onset)) periods in each channel were calculated and considered as oxy-Hb values involved in the no-go trial [6]. Finally, the changes in mean oxy-Hb levels during the no-go task under the music condition were compared with those under the no-music condition in each channel in the younger and older groups.

Statistical analysis

Shapiro-Wilk test was used to assess the distribution of age, accurate response rate, and neuropsychological test results. Data were presented as numbers and mean ± standard deviation or 95% confidence interval (95% CI) for variables with a normal distribution or median (first, third quartiles) for variables with a non-normal distribution.

The chi-square test was used to compare the ratio of participants between the two groups according to sex. Unpaired t-test or Mann-Whitney U test was used to compare the variables between the younger and older groups, as appropriate. A paired t-test or Wilcoxon signed-rank test was used to compare the accuracy response under the music and no-music conditions in the younger and older groups. To test for period and carry-over effects, the Mann-Whitney U test was used to examine the difference in and the sums of the summary scores for accuracy in the first and second sessions. One sample t-test against zero was used to assess significant changes in oxy-Hb levels during the no-go task against the go task in each channel under no-music and music conditions. A paired t-test was used to compare the oxy-Hb values under the music and no-music conditions in each group. Among the channels with a significant increase in oxy-Hb levels during the go/no-go task under the music condition compared with those under the no-music condition in the older group, the Pearson’s correlation test was applied to validate the association between changes in oxy-Hb level and changes in accurate response rates during the go/no-go task.

Statistical analyses were conducted using the IBM SPSS Statistics for Windows, Version 27 (Released 2020; IBM Corp., Armonk, New York, USA). A p-value of <0.05 indicated a statistically significant difference. P-values were corrected according to the number of NIRS channels with the Bonferroni method when analyzing the difference in oxy-Hb level changes in each channel between the no-go task and the go task under the music and no-music conditions. Bonferroni correction was also used for the results of the Shapiro-Wilk test. The correction was not applied for the abovementioned correlation tests as these correlations were exploratory [34]. The strength of the findings in terms of differences in oxy-Hb level changes under the music and no-music conditions was determined by calculating the effect size (Cohen’s d ≥ 0.2, small effect; ≥ 0.5, medium effect; and ≥ 0.8, large effect). In previous reports, the following correlation coefficients were considered: 0-0.19 as very weak; 0.20-0.39 as weak; 0.40-0.59 as moderate; 0.60-0.79 as strong; and 0.80-1 as very strong [35,36].

## Results

Characteristics of the participants

Seventy healthy adults (28 in the younger group and 42 in the older group) were enlisted. Among them, one younger individual and eight older individuals were excluded from the analysis due to the presence of motion artifacts during NIRS measurement and analytical difficulties. Two individuals in the older group were also excluded due to low scores of MMSE, HDS−R, and LM II. Finally, 27 participants with a mean age of 32 ± 8 years in the younger group and 32 participants with a mean age of 69 ± 7 years in the older group were analyzed. Table 1 shows the characteristics of the participants. The Shapiro-Wilk test revealed the normal distribution of age, LM-I, and LM-II and the non-normal distribution of the accurate response in the go/no-go test, MMSE score, HDS-R, and FAB. The ratio of participants according to sex did not significantly differ between the younger and older groups. The neuropsychological examination scores of the older group were within normal limits (Table 1).

**Table 1:** Participants’ characteristics CDR: clinical dementia rating; FAB: frontal assessment battery; HDS-R: Revised Hasegawa Dementia Scale; MMSE: Mini-Mental State Examination; WMS-R: Wechsler Memory Scale-Revised; LM I: logical memory I; LM II: logical memory II Data are presented as the number of participants, the mean ± standard deviation, or median (first and third quartiles).

Characteristic	Young	Older
n	27	32
Men, n	13	13
Women, n	14	19
Age, y	32 ± 8	69 ± 7
MMSE	-	28.5 (29.0, 30.0)
HDS-R	-	28.5 (29.0, 30.0)
FAB	-	17 (16, 18)
WMS-R LM I	-	10.0 ± 3.1
WMS-R LM II	-	9.5 ± 3.4
CDR	-	0 ± 0

Behavioral performance (accurate response rate of the go/no-go task)

Table 2 shows the accurate response rates during the go/no-go task in each condition between the younger and older groups. All errors were only observed during the go/no-go task. The older group presented with minimal but statistically significant reductions in the accurate response rate during the go/no-go task under the music condition in comparison to no-music conditions (median: 97.2% vs. 98.6%, p = 0.002). In contrast, there was no statistically significant difference in the accurate response rate in the younger group (median: 100% vs. 100%, p = 0.178). The decrease in task accuracy in the older group was statistically significant than that in the younger group (median: -1.4 vs. 0.0, p = 0.035). There were no significant period effects and carry-over effects in the healthy older adult group (p = 0.887 and 0.566, respectively) and in the younger adult group (p = 0.550 and 0.884, respectively).

**Table 2:** Accuracy response rate during the go/no-go task Data was shown as median (25, 75 percentile). ^†^p = 0.035 versus young group, ^*^p = 0.002 versus no-music in the older group

Group	No-music (%)	Music (%)	Difference
Younger	100.0 (98.6, 100.0)	100.0 (97.9, 100.0)	-0.0 (-0.7, 0.0)
Older	98.6 (95.8, 100.0)	97.2 (92.4, 98.6)^*^	-1.4 (-3.5, 0.0)^†^

Changes in oxy-Hb levels during the no-go task against the go task under the music and no-music conditions in each group

**Figure 2:**
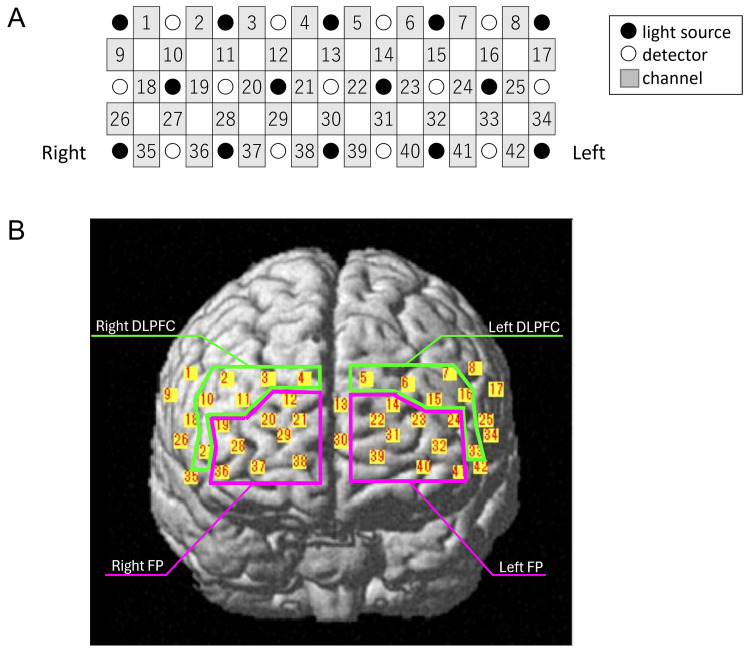
Channel location on the cortical surface. (A) Schematic illustration of the location of each optode and channel. (B) NIRS channels and regions of interest FP: frontal pole; DLPFC: dorsolateral prefrontal cortex; NIRS: near-infrared spectroscopy

**Figure 3:**
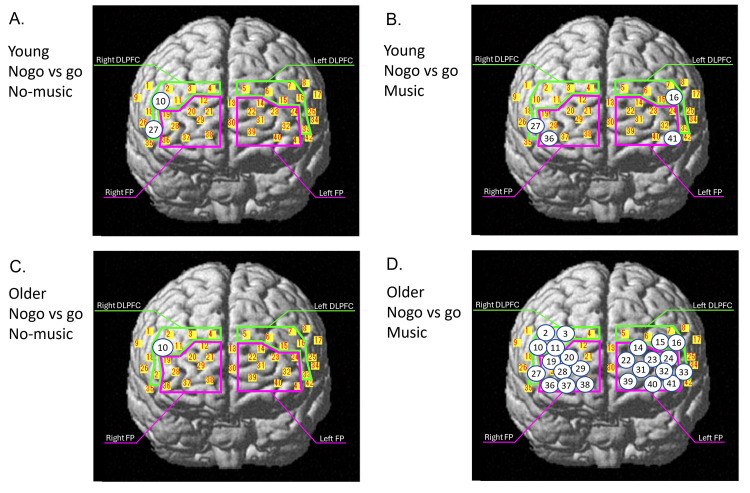
The location of the channels in which a significant difference was observed between the no-go and go tasks under no-music conditions (A) and music conditions (B) in young adults and under no-music conditions (C) and music conditions (D) in healthy older adults FP: frontal pole; DLPFC: dorsolateral prefrontal cortex

Thirty channels located in the bilateral DLPFC and FP were used in the NIRS analysis (Figure 2, Table 3). Tables 4, 5 show the oxy-Hb values between the no-go task and go task under no-music and music conditions in each channel in the younger adult (Table 4) and older adult groups (Table 5). The number of the channels, in which a significant increase in oxy-Hb levels during the no-go task against the go task was observed, increased from 2 in the right DLPFC (Ch10 and 27) under the no-music condition to 4, including 1 in the right DLPFC (Ch27), 1 in the left DLPFC (Ch16), 1 in the right FP (Ch36), and 1 in the left FP (Ch41), under the music condition in the younger adult group (Table 4, Figures 3A, 3B). Meanwhile, the number of channels increased from 1 in the right DLPFC (Ch10) to 24, including 5 in the right DLPFC (Ch2, Ch3, Ch10, Ch11, and Ch27), 3 in the left DLPFC (Ch15, Ch16, and Ch33), 7 in the right FP (Ch19, Ch20, Ch28, Ch29, Ch36, Ch37, and Ch38), and 9 in the left FP (Ch14, Ch22, Ch23, Ch24, Ch31, Ch32, Ch39, Ch40, and Ch41), in the older adult group (Table 5, Figures 3C, 3D).

**Table 3:** The location of each channel of NIRS BA: Broadman area; DLPFC: dorsolateral prefrontal cortex; FP: frontal pole; MNI: Montreal Neurological Institute coordinates; NIRS: near-infrared spectroscopy

Area	Side	Ch	MNI						BA
			X		Y		Z		
			mean	(SD)	mean	(SD)	mean	(SD)	
DLPFC	Right	2	52	(4)	24	(12)	40	(7)	BA9
		3	36	(5)	44	(9)	40	(8)	BA9
		4	17	(4)	56	(7)	41	(8)	BA9
		10	59	(4)	19	(12)	29	(5)	BA9
		11	46	(4)	44	(8)	30	(7)	BA46
		27	59	(3)	32	(9)	8	(5)	BA46
	Left	5	-6	(5)	56	(6)	42	(7)	BA9
		6	-26	(6)	49	(8)	41	(6)	BA9
		7	-44	(5)	30	(10)	42	(6)	BA9
		15	-38	(4)	48	(7)	31	(5)	BA46
		16	-53	(4)	26	(11)	31	(5)	BA9
		33	-54	(3)	36	(8)	8	(4)	BA46
FP	Right	12	27	(4)	59	(6)	30	(7)	BA10
		19	53	(4)	40	(8)	20	(6)	BA10
		20	38	(4)	59	(5)	20	(6)	BA10
		21	18	(3)	69	(3)	22	(6)	BA10
		28	46	(4)	55	(5)	9	(6)	BA10
		29	28	(4)	69	(2)	11	(6)	BA10
		36	52	(3)	48	(6)	-3	(4)	BA10
		37	38	(4)	65	(3)	-1	(5)	BA10
		38	17	(3)	73	(1)	1	(5)	BA10
	Left	14	-18	(4)	61	(5)	32	(7)	BA10
		22	-10	(5)	68	(3)	22	(7)	BA10
		23	-30	(5)	62	(5)	21	(5)	BA10
		24	-47	(4)	45	(7)	21	(5)	BA10
		31	-21	(4)	70	(2)	12	(5)	BA10
		32	-41	(4)	58	(5)	10	(5)	BA10
		39	-13	(3)	73	(1)	2	(4)	BA10
		40	-33	(4)	65	(2)	0	(4)	BA10
		41	-48	(3)	50	(5)	-2	(3)	BA10

**Table 4:** Oxy-Hb values during go task and no-go task under no-music and music condition in younger group DLPFC: dorsolateral prefrontal cortex; FP: frontal pole; SD: standard deviation; MD: mean difference; CI: confidence interval; p-value: p-value uncorrected; Sig: significance after Bonferroni correction; ns: not significant; R: right; L: left

Area	Side	CH	No-music							Music						
			Go (mM・cm)		Nogo (mM・cm)		MD (95% CI)	p-value	Sig	Go (mM・cm)		Nogo (mM・cm)		MD (95% CI)	p-value	Sig
			mean	SD	mean	SD				mean	SD	mean	SD			
DLPFC	R	2	0.001	0.008	0.009	0.053	0.009 (-0.013, 0.03)	0.413	ns	-0.001	0.01	0.015	0.054	0.017 (-0.007, 0.04)	0.152	ns
		3	0.003	0.010	0.014	0.06	0.011 (-0.013, 0.035)	0.340	ns	0.000	0.011	0.001	0.053	0.001 (-0.02, 0.023)	0.918	ns
		4	0.004	0.011	0.009	0.038	0.005 (-0.013, 0.022)	0.588	ns	0.002	0.014	0.006	0.071	0.004 (-0.025, 0.033)	0.786	ns
		10	-0.001	0.012	0.033	0.05	0.034 (0.016, 0.053)	0.001	sig	-0.003	0.008	0.025	0.057	0.028 (0.005, 0.051)	0.019	ns
		11	0.001	0.009	0.005	0.038	0.004 (-0.011, 0.019)	0.563	ns	-0.001	0.009	0.004	0.04	0.006 (-0.009, 0.02)	0.453	ns
		27	-0.002	0.009	0.044	0.063	0.046 (0.022, 0.071)	0.001	sig	0.000	0.008	0.035	0.051	0.036 (0.015, 0.056)	0.001	sig
	L	5	0.006	0.017	0.004	0.041	-0.001 (-0.017, 0.014)	0.845	ns	0.000	0.016	0.008	0.041	0.007 (-0.013, 0.027)	0.463	ns
		6	-0.002	0.009	0.013	0.072	0.015 (-0.021, 0.051)	0.398	ns	0.000	0.017	0.017	0.087	0.017 (-0.032, 0.066)	0.48	ns
		7	0.003	0.010	0.016	0.073	0.013 (-0.016, 0.042)	0.37	ns	-0.003	0.011	0.009	0.069	0.013 (-0.018, 0.043)	0.397	ns
		15	0.000	0.013	0.010	0.083	0.01 (-0.021, 0.042)	0.499	ns	-0.001	0.011	0.018	0.053	0.02 (-0.001, 0.04)	0.063	ns
		16	0.002	0.012	0.013	0.076	0.011 (-0.018, 0.04)	0.443	ns	0.001	0.011	0.027	0.038	0.025 (0.011, 0.04)	0.001	sig
		33	-0.001	0.008	0.030	0.076	0.031 (0.001, 0.06)	0.043	ns	0.000	0.01	0.03	0.045	0.03 (0.012, 0.048)	0.002	ns
FP	R	12	0.000	0.008	0.011	0.043	0.011 (-0.006, 0.027)	0.196	ns	0.001	0.008	0.009	0.04	0.008 (-0.009, 0.024)	0.350	ns
		19	-0.001	0.009	0.020	0.052	0.021 (0.002, 0.041)	0.033	ns	-0.002	0.01	0.012	0.042	0.014 (-0.003, 0.031)	0.098	ns
		20	-0.001	0.010	0.010	0.044	0.011 (-0.007, 0.028)	0.21	ns	0.000	0.01	0.009	0.038	0.008 (-0.006, 0.023)	0.251	ns
		21	0.000	0.010	0.011	0.045	0.011 (-0.007, 0.03)	0.219	ns	0.000	0.008	0.009	0.032	0.009 (-0.004, 0.021)	0.156	ns
		28	-0.001	0.012	0.015	0.057	0.016 (-0.007, 0.039)	0.161	ns	0.000	0.013	0.024	0.051	0.025 (0.005, 0.044)	0.018	ns
		29	0.003	0.016	0.005	0.058	0.003 (-0.022, 0.027)	0.822	ns	0.002	0.014	0.021	0.054	0.019 (-0.003, 0.04)	0.085	ns
		36	-0.001	0.010	0.030	0.055	0.031 (0.009, 0.053)	0.007	ns	0.001	0.008	0.047	0.044	0.046 (0.028, 0.063)	0.000	sig
		37	0.001	0.016	0.015	0.066	0.014 (-0.015, 0.044)	0.328	ns	0.000	0.011	0.03	0.055	0.03 (0.008, 0.052)	0.008	ns
		38	0.002	0.012	0.006	0.062	0.004 (-0.022, 0.03)	0.741	ns	0.000	0.008	0.017	0.044	0.017 (0, 0.035)	0.052	ns
	L	14	0.001	0.010	0.000	0.092	-0.001 (-0.037, 0.035)	0.957	ns	0.000	0.011	0.02	0.061	0.02 (-0.005, 0.044)	0.118	ns
		22	0.001	0.008	0.005	0.042	0.004 (-0.014, 0.022)	0.641	ns	0.002	0.007	0.002	0.039	0 (-0.014, 0.015)	0.958	ns
		23	0.001	0.009	0.016	0.070	0.015 (-0.013, 0.044)	0.273	ns	0.002	0.01	0.007	0.054	0.005 (-0.016, 0.026)	0.638	ns
		24	-0.001	0.010	0.016	0.085	0.017 (-0.016, 0.05)	0.295	ns	-0.001	0.011	0.013	0.033	0.014 (0, 0.028)	0.050	ns
		31	0.004	0.012	0.007	0.070	0.004 (-0.025, 0.032)	0.803	ns	0.002	0.012	0.012	0.056	0.01 (-0.012, 0.032)	0.374	ns
		32	-0.002	0.014	0.015	0.075	0.018 (-0.013, 0.048)	0.242	ns	0.000	0.012	0.02	0.058	0.02 (-0.002, 0.042)	0.076	ns
		39	0.001	0.011	0.016	0.056	0.015 (-0.008, 0.038)	0.182	ns	0.002	0.01	0.023	0.045	0.021 (0.003, 0.038)	0.021	ns
		40	0.000	0.013	0.016	0.072	0.017 (-0.012, 0.046)	0.25	ns	-0.001	0.013	0.029	0.062	0.031 (0.006, 0.055)	0.017	ns
		41	-0.001	0.009	0.038	0.077	0.039 (0.009, 0.069)	0.012	ns	0.000	0.009	0.043	0.055	0.043 (0.021, 0.065)	0.000	sig

**Table 5:** Oxy-Hb values during go task and no-go task under no-music and music condition in healthy older group DLPFC: dorsolateral prefrontal cortex; FP: frontal pole; SD: standard deviation; MD: mean difference; CI: confidence interval; p-value: p-value uncorrected; Sig: significance after Bonferroni correction; ns: not significant; R: right; L: left

Area	Side	Ch	No-music							Music						
			Go (mM・cm)		Nogo (mM・cm)		MD (95% CI)	p-value	Sig	Go (mM・cm)		Nogo (mM・cm)		MD (95% CI)	p-value	Sig
			mean	SD	mean	SD				mean	SD	mean	SD			
DLPFC	R	2	-0.001	0.009	0.008	0.042	0.009 (-0.006, 0.025)	0.217	ns	-0.002	0.008	0.029	0.036	0.031 (0.017, 0.045)	0.000	sig
		3	0.000	0.007	-0.008	0.034	-0.008 (-0.02, 0.005)	0.213	ns	-0.002	0.007	0.022	0.038	0.024 (0.011, 0.038)	0.001	sig
		4	-0.002	0.007	0.010	0.031	0.012 (-0.001, 0.024)	0.062	ns	-0.002	0.006	0.009	0.036	0.011 (-0.002, 0.024)	0.087	ns
		10	-0.003	0.012	0.027	0.046	0.031 (0.013, 0.048)	0.001	sig	-0.004	0.007	0.042	0.042	0.046 (0.03, 0.061)	0.000	sig
		11	0.000	0.009	0.002	0.051	0.002 (-0.017, 0.02)	0.870	ns	-0.003	0.008	0.04	0.051	0.043 (0.024, 0.062)	0.000	sig
		27	-0.002	0.008	0.028	0.057	0.03 (0.009, 0.051)	0.007	ns	-0.006	0.008	0.05	0.052	0.056 (0.037, 0.075)	0.000	sig
	L	5	-0.002	0.006	-0.003	0.028	-0.001 (-0.015, 0.012)	0.832	ns	-0.003	0.005	0.005	0.025	0.007 (-0.005, 0.02)	0.219	ns
		6	0.000	0.009	0.000	0.049	-0.001 (-0.018, 0.017)	0.928	ns	-0.003	0.007	0.023	0.046	0.026 (0.008, 0.043)	0.005	ns
		7	-0.002	0.01	0.003	0.037	0.005 (-0.01, 0.019)	0.506	ns	-0.003	0.008	0.021	0.073	0.024 (-0.001, 0.05)	0.057	ns
		15	0.000	0.009	0.009	0.045	0.01 (-0.006, 0.025)	0.215	ns	-0.004	0.008	0.041	0.049	0.045 (0.027, 0.064)	0.000	sig
		16	-0.001	0.01	0.003	0.041	0.004 (-0.01, 0.018)	0.542	ns	-0.003	0.011	0.041	0.05	0.044 (0.026, 0.062)	0.000	sig
		33	-0.002	0.008	0.027	0.06	0.029 (0.006, 0.052)	0.014	ns	-0.003	0.007	0.043	0.042	0.046 (0.031, 0.061)	0.000	sig
FP	R	12	0.000	0.006	-0.003	0.035	-0.003 (-0.015, 0.01)	0.640	ns	-0.002	0.006	0.022	0.043	0.024 (0.007, 0.041)	0.007	ns
		19	-0.001	0.01	0.010	0.061	0.011 (-0.011, 0.033)	0.296	ns	-0.005	0.008	0.044	0.057	0.049 (0.027, 0.07)	0.000	sig
		20	0.000	0.009	0.007	0.044	0.006 (-0.01, 0.023)	0.433	ns	-0.003	0.007	0.034	0.046	0.037 (0.019, 0.055)	0.000	sig
		21	0.000	0.008	0.000	0.036	-0.001 (-0.014, 0.012)	0.930	ns	-0.002	0.006	0.02	0.044	0.023 (0.006, 0.039)	0.009	ns
		28	-0.001	0.014	0.015	0.069	0.015 (-0.01, 0.041)	0.222	ns	-0.006	0.011	0.052	0.054	0.057 (0.036, 0.078)	0.000	sig
		29	0.001	0.012	0.001	0.049	0 (-0.018, 0.018)	0.975	ns	-0.003	0.007	0.036	0.054	0.039 (0.019, 0.06)	0.000	sig
		36	0.000	0.009	0.018	0.051	0.019 (0, 0.038)	0.055	ns	-0.005	0.007	0.047	0.047	0.052 (0.035, 0.07)	0.000	sig
		37	0.000	0.011	0.001	0.052	0.001 (-0.019, 0.02)	0.933	ns	-0.004	0.011	0.057	0.057	0.062 (0.04, 0.084)	0.000	sig
		38	0.000	0.01	-0.016	0.046	-0.016 (-0.035, 0.003)	0.094	ns	-0.004	0.009	0.04	0.049	0.044 (0.025, 0.063)	0.000	sig
	L	14	-0.001	0.009	-0.002	0.044	-0.001 (-0.017, 0.015)	0.902	ns	-0.001	0.006	0.038	0.051	0.039 (0.02, 0.058)	0.000	sig
		22	0.000	0.008	-0.004	0.036	-0.004 (-0.017, 0.009)	0.550	ns	-0.002	0.007	0.025	0.039	0.027 (0.012, 0.042)	0.001	sig
		23	-0.001	0.009	0.007	0.040	0.008 (-0.007, 0.022)	0.283	ns	-0.002	0.007	0.038	0.044	0.04 (0.024, 0.055)	0.000	sig
		24	-0.001	0.009	0.005	0.035	0.006 (-0.007, 0.018)	0.387	ns	-0.003	0.008	0.033	0.038	0.036 (0.022, 0.05)	0.000	sig
		31	0.000	0.01	0.006	0.052	0.006 (-0.012, 0.025)	0.497	ns	-0.004	0.007	0.047	0.045	0.051 (0.035, 0.067)	0.000	sig
		32	-0.002	0.013	0.015	0.044	0.017 (0.001, 0.033)	0.041	ns	-0.005	0.008	0.055	0.046	0.059 (0.043, 0.076)	0.000	sig
		39	0.000	0.009	-0.010	0.042	-0.01 (-0.027, 0.007)	0.225	ns	-0.004	0.01	0.043	0.047	0.047 (0.029, 0.065)	0.000	sig
		40	0.000	0.011	0.010	0.047	0.01 (-0.007, 0.027)	0.252	ns	-0.005	0.009	0.051	0.051	0.056 (0.038, 0.074)	0.000	sig
		41	-0.001	0.01	0.027	0.049	0.028 (0.01, 0.047)	0.004	ns	-0.005	0.007	0.044	0.049	0.049 (0.032, 0.066)	0.000	sig

Changes in oxy-Hb levels during the go/no-go tasks between the music and no-music conditions

**Figure 4:**
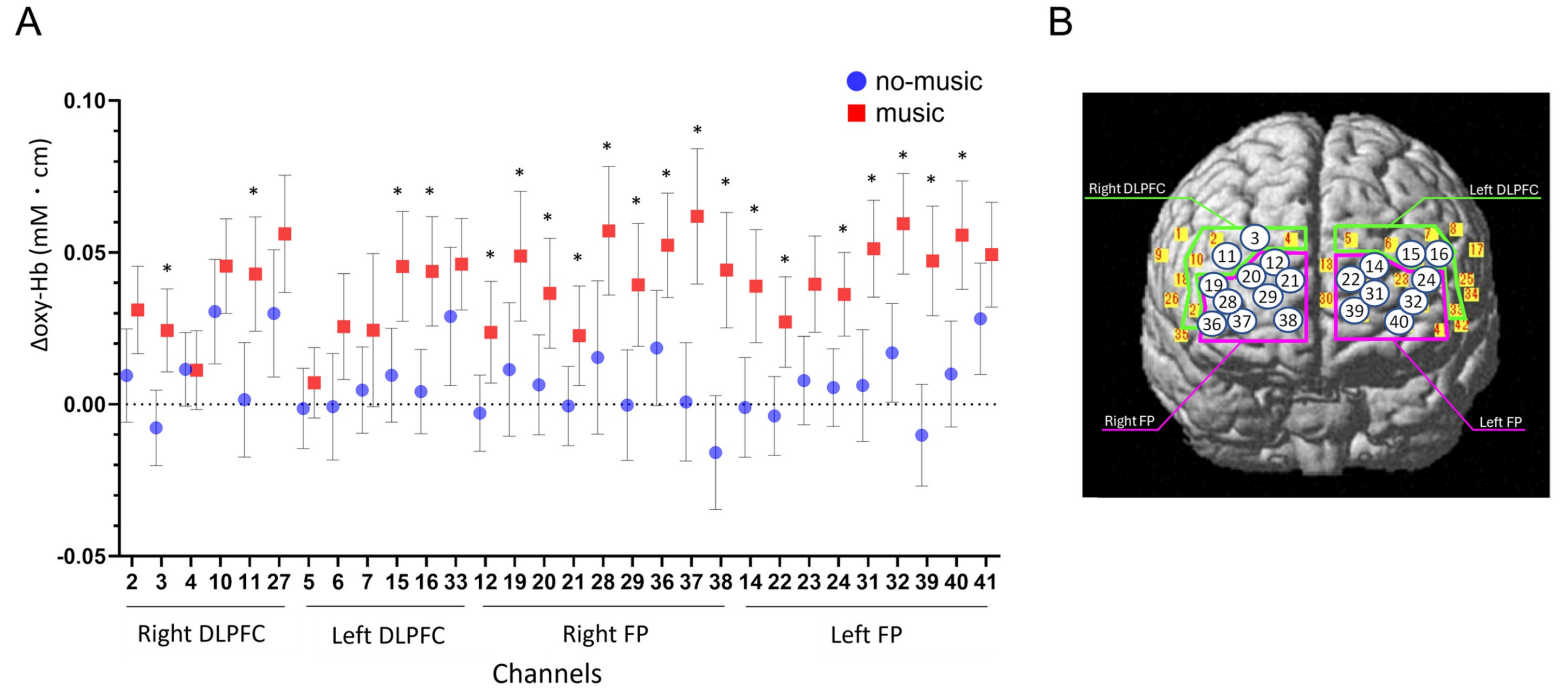
(A) Changes in oxy-Hb levels under the music and no-music conditions during the go/no-go task from the go task in the older group. (B) The location of the channels in which significant increases in oxy-Hb levels were observed under the music condition in older adults. The numbers in the circle indicate the channels DLPFC: dorsolateral prefrontal cortex; FP: frontal pole; oxy-Hb: oxyhemoglobin Error bars indicate 95% CI. ^*^p < 0.05 adjusted using the Bonferroni method versus no-music condition.

**Figure 5:**
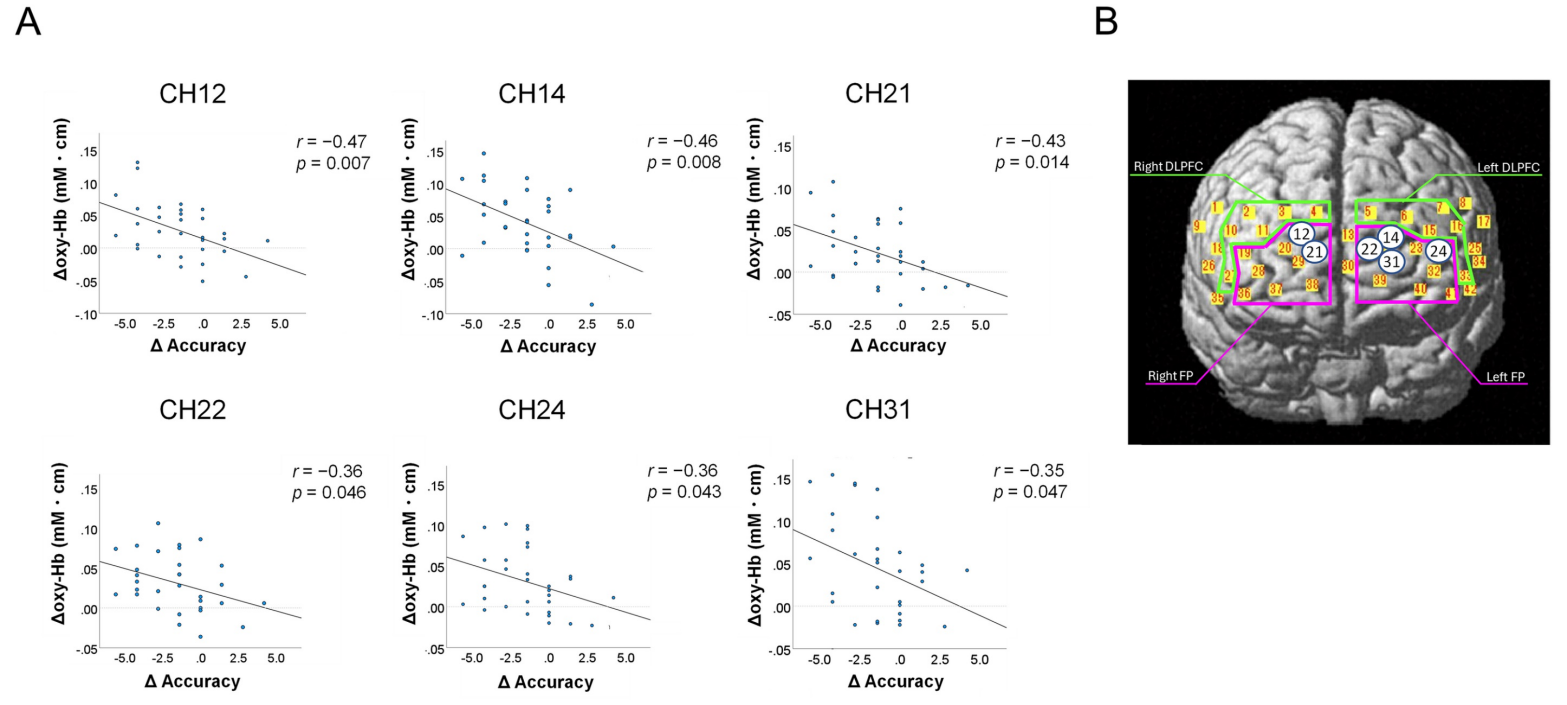
(A) The significant negative correlation between changes in the task accuracy and changes in oxy-Hb levels under the music condition in the six channels in older adults. (B) The location of the channels in which a significant correlation between the decline in the task accuracy and increased oxy-Hb levels under the music condition were observed in older adults DLPFC: dorsolateral prefrontal cortex; FP: frontal pole; oxy-Hb: oxyhemoglobin The numbers in the circle indicate the channels.

In the younger group, there were no significant differences in the increase in oxy-Hb levels between music and no-music conditions in all channels (Table 6). The increase in oxy-Hb levels in most channels in the older group was more likely to be greater under the music condition than under the no-music condition (Table 7). In the older group, moreover, the differences in oxy-Hb level changes in 20 channels located in the bilateral BA9 and BA46 (DLPFC) and the bilateral BA10 (FP) under the music and no-music conditions were statistically significant, and medium- or large-size effects were detected in these channels (Table 7, Figure 4). In six channels, including two channels in the right BA10 (Ch12 and 21) and four channels in the left BA10 (Ch14, 22, 24, and 31), the greater decline in the accurate response rate under the music condition was significantly correlated with a higher increase in oxy-Hb levels during the go/no-go task in the older group (Table 8, Figure 5). The correlation coefficient was 0.35-0.47, which indicated a weak to moderate correlation.

**Table 6:** Oxy-Hb changes during go/no-go task under music and no-music conditions in younger group DLPFC: dorsolateral prefrontal cortex; FP: frontal pole; SD: standard deviation; MD: mean difference; CI: confidence interval; p-value: p-value uncorrected; Sig: significance after Bonferroni correction; ns: not significant; R: right; L: left

Area	Side	CH	Music (mM・cm) (mean ± SD)	No-music (mM・cm) (mean ± SD)	MD (95% CI)	p-value	Sig	Cohen's d	Effect size
DLPFC	R	2	0.017 ± 0.057	0.009 ± 0.054	0.008 (-0.022, 0.037)	0.592	ns	0.14	no
		3	0.001 ± 0.052	0.011 ± 0.058	-0.010 (-0.037, 0.017)	0.443	ns	-0.19	no
		4	0.004 ± 0.066	0.005 ± 0.04	-0.001 (-0.03, 0.028)	0.954	ns	-0.02	no
		10	0.028 ± 0.058	0.034 ± 0.047	-0.006 (-0.034, 0.022)	0.670	ns	-0.11	no
		11	0.006 ± 0.038	0.004 ± 0.037	0.001 (-0.021, 0.024)	0.907	ns	0.04	no
		27	0.036 ± 0.052	0.046 ± 0.062	-0.011 (-0.042, 0.021)	0.500	ns	-0.18	no
	L	5	0.007 ± 0.044	-0.001 ± 0.034	0.009 (-0.012, 0.03)	0.400	ns	0.22	small
		6	0.017 ± 0.098	0.015 ± 0.072	0.002 (-0.044, 0.048)	0.928	ns	0.02	no
		7	0.013 ± 0.073	0.013 ± 0.071	0.000 (-0.037, 0.037)	0.982	ns	-0.01	no
		15	0.020 ± 0.052	0.010 ± 0.079	0.009 (-0.029, 0.047)	0.631	ns	0.14	no
		16	0.025 ± 0.037	0.011 ± 0.073	0.015 (-0.014, 0.043)	0.300	ns	0.24	small
		33	0.030 ± 0.046	0.031 ± 0.075	-0.001 (-0.033, 0.031)	0.957	ns	-0.01	no
FP	R	12	0.008 ± 0.041	0.011 ± 0.042	-0.003 (-0.026, 0.02)	0.776	ns	-0.08	no
		19	0.014 ± 0.043	0.021 ± 0.049	-0.007 (-0.031, 0.017)	0.533	ns	-0.16	no
		20	0.008 ± 0.037	0.011 ± 0.044	-0.003 (-0.027, 0.022)	0.831	ns	-0.06	no
		21	0.009 ± 0.031	0.011 ± 0.047	-0.003 (-0.024, 0.019)	0.809	ns	-0.07	no
		28	0.025 ± 0.05	0.016 ± 0.058	0.008 (-0.02, 0.037)	0.551	ns	0.16	no
		29	0.019 ± 0.054	0.003 ± 0.062	0.016 (-0.013, 0.045)	0.270	ns	0.27	small
		36	0.046 ± 0.045	0.031 ± 0.055	0.015 (-0.012, 0.042)	0.275	ns	0.29	small
		37	0.030 ± 0.055	0.014 ± 0.074	0.016 (-0.024, 0.056)	0.425	ns	0.24	small
		38	0.017 ± 0.044	0.004 ± 0.066	0.013 (-0.019, 0.045)	0.411	ns	0.23	small
	L	14	0.020 ± 0.061	-0.001 ± 0.09	0.021 (-0.029, 0.07)	0.398	ns	0.27	small
		22	0.000 ± 0.036	0.004 ± 0.044	-0.004 (-0.025, 0.018)	0.725	ns	-0.09	no
		23	0.005 ± 0.053	0.015 ± 0.071	-0.011 (-0.046, 0.025)	0.547	ns	-0.17	no
		24	0.014 ± 0.035	0.017 ± 0.083	-0.003 (-0.037, 0.031)	0.849	ns	-0.05	no
		31	0.010 ± 0.056	0.004 ± 0.073	0.006 (-0.027, 0.039)	0.700	ns	0.1	no
		32	0.020 ± 0.056	0.018 ± 0.076	0.002 (-0.038, 0.043)	0.901	ns	0.04	no
		39	0.021 ± 0.044	0.015 ± 0.057	0.006 (-0.022, 0.033)	0.670	ns	0.11	no
		40	0.031 ± 0.062	0.017 ± 0.074	0.014 (-0.024, 0.052)	0.459	ns	0.21	small
		41	0.043 ± 0.056	0.039 ± 0.075	0.004 (-0.026, 0.034)	0.782	ns	0.06	no

**Table 7:** Oxy-Hb changes during go/no-go task under music and no-music conditions in older group DLPFC: dorsolateral prefrontal cortex; FP: frontal pole; SD: standard deviation; MD: mean difference; CI: confidence interval; p-value: p-value uncorrected; Sig: significance after Bonferroni correction; ns: not significant; R: right; L: left

Area	Side	CH	Music (mM・cm) (mean ± SD)	No-music (mM・cm) (mean ± SD)	MD (95% CI)	p-value	Sig	Cohen‘s d	Effect size
DLPFC	R	2	0.031 ± 0.039	0.009 ± 0.042	0.022 (0.004, 0.04)	0.021	ns	0.53	medium
		3	0.024 ± 0.037	-0.008 ± 0.034	0.032 (0.016, 0.048)	0.000	sig	0.90	large
		4	0.011 ± 0.035	0.012 ± 0.033	0.000 (-0.02, 0.019)	0.976	ns	-0.01	no
		10	0.046 ± 0.042	0.031 ± 0.046	0.015 (-0.005, 0.035)	0.130	ns	0.34	small
		11	0.043 ± 0.052	0.002 ± 0.052	0.041 (0.021, 0.062)	0.000	sig	0.79	medium
		27	0.056 ± 0.052	0.030 ± 0.056	0.026 (0.008, 0.044)	0.006	ns	0.48	small
	L	5	0.007 ± 0.027	-0.001 ± 0.030	0.009 (-0.006, 0.024)	0.231	ns	0.31	small
		6	0.026 ± 0.048	-0.001 ± 0.049	0.026 (0.007, 0.046)	0.011	ns	0.54	medium
		7	0.024 ± 0.07	0.005 ± 0.039	0.02 (-0.007, 0.046)	0.137	ns	0.34	small
		15	0.045 ± 0.05	0.01 ± 0.043	0.036 (0.016, 0.056)	0.001	sig	0.77	medium
		16	0.044 ± 0.049	0.004 ± 0.038	0.04 (0.022, 0.058)	0.000	sig	0.89	large
		33	0.046 ± 0.04	0.029 ± 0.061	0.017 (-0.006, 0.04)	0.141	ns	0.33	small
FP	R	12	0.024 ± 0.046	-0.003 ± 0.034	0.027 (0.011, 0.042)	0.002	sig	0.65	medium
		19	0.049 ± 0.059	0.011 ± 0.061	0.037 (0.016, 0.059)	0.001	sig	0.62	medium
		20	0.037 ± 0.050	0.006 ± 0.046	0.03 (0.015, 0.046)	0.000	sig	0.63	medium
		21	0.023 ± 0.046	-0.001 ± 0.036	0.023 (0.01, 0.036)	0.001	sig	0.55	medium
		28	0.057 ± 0.059	0.015 ± 0.070	0.042 (0.019, 0.065)	0.001	sig	0.64	medium
		29	0.039 ± 0.056	0.000 ± 0.050	0.04 (0.022, 0.057)	0.000	sig	0.74	medium
		36	0.052 ± 0.047	0.019 ± 0.052	0.034 (0.016, 0.052)	0.001	sig	0.68	medium
		37	0.062 ± 0.062	0.001 ± 0.054	0.061 (0.043, 0.079)	0.000	sig	1.04	large
		38	0.044 ± 0.049	-0.016 ± 0.048	0.06 (0.041, 0.079)	0.000	sig	1.23	large
	L	14	0.039 ± 0.052	-0.001 ± 0.046	0.04 (0.021, 0.058)	0.000	sig	0.82	large
		22	0.027 ± 0.041	-0.004 ± 0.035	0.031 (0.018, 0.044)	0.000	sig	0.81	large
		23	0.040 ± 0.044	0.008 ± 0.040	0.032 (0.012, 0.052)	0.003	ns	0.75	medium
		24	0.036 ± 0.038	0.006 ± 0.035	0.031 (0.017, 0.044)	0.000	sig	0.83	large
		31	0.051 ± 0.044	0.006 ± 0.051	0.045 (0.024, 0.066)	0.000	sig	0.94	large
		32	0.059 ± 0.046	0.017 ± 0.045	0.043 (0.022, 0.063)	0.000	sig	0.93	large
		39	0.047 ± 0.048	-0.010 ± 0.044	0.057 (0.038, 0.077)	0.000	sig	1.25	large
		40	0.056 ± 0.049	0.010 ± 0.048	0.046 (0.026, 0.066)	0.000	sig	0.94	large
		41	0.049 ± 0.047	0.028 ± 0.050	0.021 (0.002, 0.04)	0.028	ns	0.43	small

**Table 8:** Correlation between the changes in the accuracy of go/no-go test and oxy-Hb levels in 20 channels in which significant changes were observed between the music and no-music conditions in older adults DLPFC: dorsolateral prefrontal cortex; FP: frontal pole; oxy-Hb: oxyhemoglobin; Sig: significance; ns: not significant

Area	Side	CH	Pearson r	95% CI	p-value	Sig
DLPFC	R	3	0.105	-0.259, 0.443	0.574	ns
		11	-0.144	-0.469, 0.215	0.431	ns
	L	15	-0.293	-0.583, 0.062	0.103	ns
		16	-0.21	-0.526, 0.155	0.256	ns
FP	R	12	-0.473	-0.708, -0.142	0.007	sig
		19	-0.16	-0.482, 0.199	0.381	ns
		20	-0.16	-0.482, 0.2	0.382	ns
		21	-0.429	-0.676, -0.094	0.014	sig
		28	0.043	-0.31, 0.386	0.815	ns
		29	-0.258	-0.556, 0.1	0.154	ns
		36	-0.114	-0.45, 0.25	0.541	ns
		37	-0.217	-0.526, 0.142	0.233	ns
		38	-0.31	-0.612, 0.071	0.109	ns
	L	14	-0.461	-0.698, -0.134	0.008	sig
		22	-0.361	-0.634, -0.007	0.046	sig
		24	-0.361	-0.63, -0.014	0.043	sig
		31	-0.353	-0.625, -0.005	0.047	sig
		32	-0.249	-0.55, 0.109	0.169	ns
		39	-0.32	-0.614, 0.053	0.091	ns
		40	-0.316	-0.599, 0.037	0.078	ns

## Discussion

This report demonstrated that background music during an inhibition task decreased inhibition control and significantly increased PFC activity in DLPFC and FP in the healthy older group but not in the young group. Furthermore, a significant negative correlation was observed between increased oxy-Hb levels in the FP and the decline in the accuracy response rate in the older group.

The present study showed that background music had a negative effect on cognitive task performance in the healthy older group but not in the younger group. Previous studies have assessed the negative effect of music on cognitive task performance and shown that relaxing music interferes with attentional control compared with stimulating music and silence [11]. Music has a distracting effect on novice surgeons performing new tasks in the operation theater [10]. Designers showed an improvement in their performance in alerting and orienting attention under no-music conditions compared with that under music conditions, indicating that an environment with music decreases the concentration of designers [37].

The negative effect of background music on cognitive tasks was observed in not younger but older groups in the present study. In contrast to the findings of the present study, some studies have shown no difference between younger and older adults in terms of the influences of music on attentional control [11,16]. Some studies using other cognitive tasks, such as memory performance, have shown that the executive function of older adults, compared with that of younger adults, declined due to music [15,38]. EI Haj et al. reported that older adults had a significant reduction in source memory after music exposure compared with young adults [38]. Reaves et al. showed that background music had impaired visual associative memory performance compared with silence in older adults but not in younger ones [15]. Given these findings, older adults might want to avoid listening to music when performing tasks that require intense attention.

Our findings revealed a significant increase in oxy-Hb levels in the PFC, which is associated with attention control, inhibition control, and dual-task performance [17-20], in the healthy older group but not in the younger group. Furthermore, a significant negative correlation was observed between increased oxy-Hb levels in the FP and the decline in accuracy response rate under the music condition in the older group. Previous studies have revealed that older adults exhibit greater brain activities than young adults during the selective inhibition task in the NIRS study [23,24]. In an fMRI study using the go/nogo task, older adults showed more brain activity during the task compared to young adults, and the increases in brain activation were related to good inhibitory performance [39]. An fMRI study using an antisaccade task revealed that older adults, not young ones, showed additional recruitment of the FP, which was correlated with faster antisaccade reaction times, and increased DLPFC activity, which was associated with better performance in inhibitory control [40]. Increased PFC activation in older adults has been explained as compensation for the reduced executive function [41,42]. It has also been explained using posterior to anterior sift theory in aging [43]. Meanwhile, increased PFC activation accompanied by aging reflects a less specific or efficient activity rather than compensation [44]. The present study revealed a negative correlation between PFC activity and response accuracy, thereby indicating that increased PFC activity was related to reduced response accuracy, which might be reflected as confused cognitive function due to background music in older adults.

Whether the music was specific for the impairment effect of inhibition control and PFC activation was unclear. The current study aimed to investigate the difference between with and without music. A previous study revealed that classical music does not affect cognitive functions, whereas white noise impairs these functions [45]. Another study showed that exposure to music, but not silence or street noise, interferes with cognitive functions, such as working memory [38]. Further research using control conditions with different types of music or sounds (e.g., white noise) must be conducted to determine the effect of different types of music or sounds on inhibition control and PFC activation in older adults.

The type of music and task difficulties might be associated with task performance decline while listening to background music. Music has several components, including tempo, rhythm, melody, lyrics, and mode, such as exciting, happy, and sad. Relaxing music has interfered with visuospatial attention [11]. The tempo of background music can influence concentration and performance [12,13,46]. Dovorany et al. reported that music with the major mode using a fast tempo and minor mode using a slow tempo modulated different aspects of attention after listening to music [46]. Music with a speed-up tempo can make drivers more excited and decrease their concentration, which leads to a less stable performance [12]. Listening to fast-tempo music is associated with increased mental load and reduced hazard perception ability during traffic among novice drivers; meanwhile, listening to slow-tempo music does not increase the mental load of novice drivers and can have some benefits to hazard perception [13]. The current study could not validate the influences of melody tempo because it was set at a stable value of 120 bpm. Further studies using various tempos of melody should be performed.

A previous study showed that preferred background music can enhance task-focused attentional status on a low-demanding sustained-attention task in students [8]. Qiu et al. have revealed that music promotes brain activity, particularly in the PFC, and the activation induced by personally preferred music is more robust than that of neutral music in an EEG-fNIRS study [47]. In the present study, we did not investigate the degree of preference for the music. Further research should be performed to validate the effects of music on executive function and how PFC activity can be influenced by music preference in older adults.

Herein, the inhibitory control and PFC activation differed between healthy younger and older adults based on an analysis using a relatively simple, noninvasive method. Some types of go/no-go tasks were used in the previous studies [5-7,48]. The go/no-go task using color was simpler than that using images such as animal figures. NIRS is a noninvasive technique to monitor cerebral activity and is associated with less physical restriction than fMRI. Reportedly, a decline in attention and inhibitory control can predict future accidents in older adults [3,4]. Higher levels of PFC activation during the dual task can predict falls in older adults [49]. Herein, performing tasks while listening to background music might work as a dual task and interfere with task performance. Therefore, a decline in inhibitory task performance and an increase in PFC activity while listening to background music may predict future accidents in older adults. Further studies must evaluate the association between a decline in inhibitory control and an increase in PFC activity, as observed in older adults herein, as well as future accidents.

This study had some limitations in terms of result interpretation. That is, the degree of concentration for listening to background music could not be quantified, although the participants were instructed to listen to it carefully under the music condition. As mentioned above, whether other types of music or tempo influence the inhibition of control performance and PFC activation remains unclear. Further research should be performed to validate these issues.

## Conclusions

Listening to background music reduced the accuracy of inhibition task responses in older but not younger people. It also increased the PFC activation associated with attention and inhibitory function. Thus, background music is an interference stimulus, and it can decrease attention and significantly increase PFC activity in older adults. Older people might want to avoid listening to music when performing tasks requiring strong attention. Further large-scale cohort studies must be performed to validate our results.
